# 2208. Ten years of the Irish National Outpatient Parenteral Antimicrobial Therapy (OPAT) Registry Data

**DOI:** 10.1093/ofid/ofad500.1830

**Published:** 2023-11-27

**Authors:** Paul Reidy, Eavan Muldoon

**Affiliations:** Trinity College Dublin, Dublin, Dublin, Ireland; Mater Misericordiae University Hospital/University College Dublin/Health Services Executive, Dublin, Dublin, Ireland

## Abstract

**Background:**

The Irish Outpatient Parenteral Antimicrobial Therapy (OPAT) programme, a national centrally administered outpatient antibiotic provision service was established in 2013 within the public hospital system and is operated by the Health Service Executive (HSE). Ireland is relatively unique in having a nationally coordinated OPAT programme with complete summary data since its establishment.

Here we describe the programme usage over ten years and show the data can be used to identify important trends in national antimicrobial usage, including stewardship issues, with relevant lessons for other OPAT centres.

**Methods:**

Using data extracted from the national electronic portal we describe summary statistics from January 2013 to December 2022 (10 years).

**Results:**

Over the ten-year period 17,559 OPAT episodes were facilitated across 42 healthcare institutions. These episodes represented 14,955 unique care episodes with the remainder being extensions or changes to active episodes.

12388 episodes were home OPAT delivered by a healthcare worker versus 5170 self OPAT where patients were taught to self-administer the antimicrobials.

The median OPAT duration of therapy was 14 days with an average of 19.9 days.

The median age of a referred patient was 59 years with an average age of 56 years.

The top five diagnoses of osteomyelitis, abscesses, cellulitis, bacteraemia, and pyelonephritis accounted for 53% of all referrals.

81.5% of patients were prescribed a single antimicrobial, 17.9% two agents and 0.6% three.

Four antibiotics – ceftriaxone, daptomycin, cefazolin and flucloxacillin continuous infusion account of 59% of the 44 different antimicrobials prescribed via the programme.

Five of the 42 healthcare institutions accounted for 57.25% of all OPAT prescriptions. The number of beds in a hospital was not the strongest predictor of OPAT usage.

The total number of bed days saved in the 10-year period was 292,860.

**Figure:**
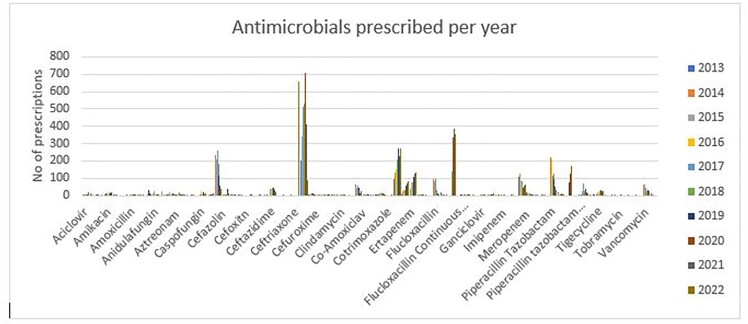

**Figure:**
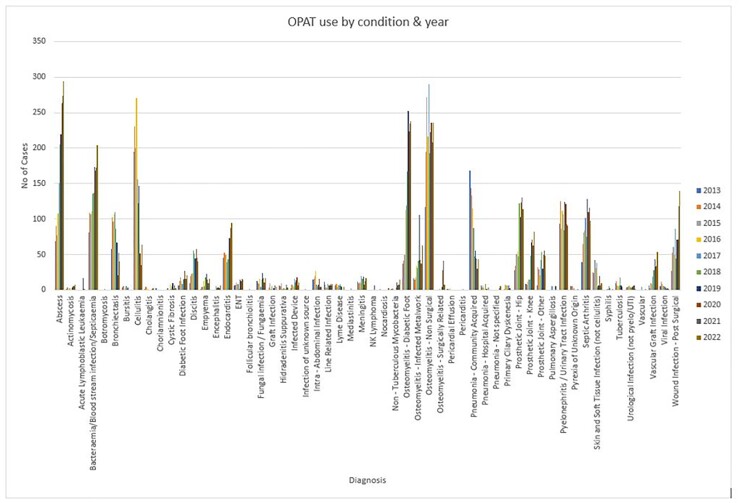

**Figure:**
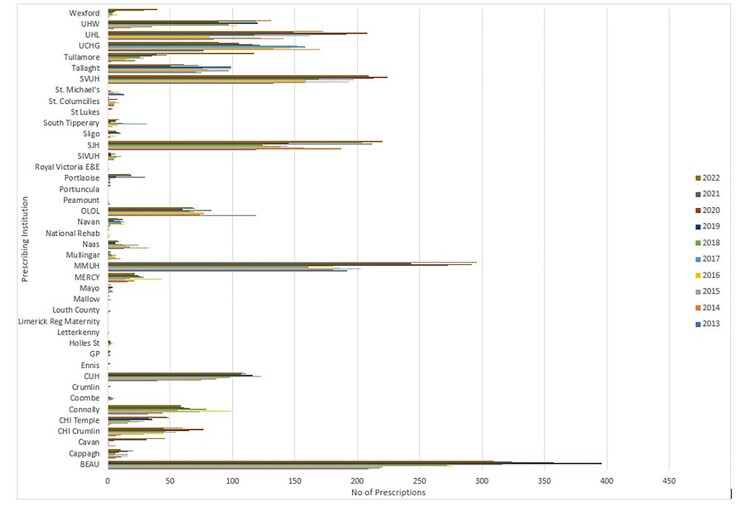

**Conclusion:**

Within a health system operating on 11,171 beds available per day, for a population of just over 5 million people, with an average daily running cost of €878 per acute bed, the National OPAT programme has already contributed to significant bed day and consequent financial savings.

Starting an infectious diseases clinical service significantly increases OPAT utilisation rates.

**Figure:**
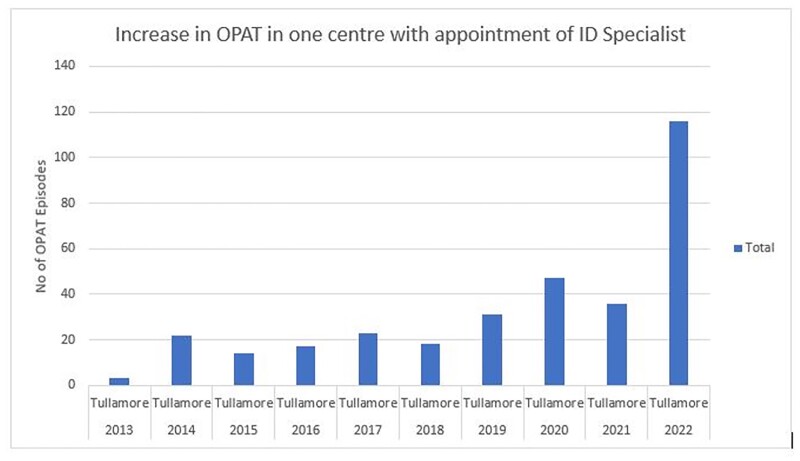

ID Specialist appointed to centre for first time in 2021

**Disclosures:**

**All Authors**: No reported disclosures

